# Long non-coding RNAs in cancer progression

**DOI:** 10.3389/fgene.2012.00219

**Published:** 2012-10-24

**Authors:** Keiko Tano, Nobuyoshi Akimitsu

**Affiliations:** Radioisotope Center, The University of TokyoTokyo, Japan

**Keywords:** large non-coding RNA, cancer, disease, MALAT1, HOTAIR, ANRIL

## Abstract

Recent large-scale transcriptome analyses have revealed that transcription is spread throughout the mammalian genomes, yielding large numbers of transcripts, including long non-coding RNAs (lncRNAs) with little or no protein-coding capacity. Dozens of lncRNAs have been identified as biologically significant. In many cases, lncRNAs act as key molecules in the regulation of processes such as chromatin remodeling, transcription, and post-transcriptional processing. Several lncRNAs (e.g., MALAT1, HOTAIR, and ANRIL) are associated with human diseases, including cancer. Those lncRNAs associated with cancer are often aberrantly expressed. Although the underlying molecular mechanisms by which lncRNAs regulate cancer development are unclear, recent studies have revealed that such aberrant expression of lncRNAs affects the progression of cancers. In this review, we highlight recent findings regarding the roles of lncRNAs in cancer biology.

## INTRODUCTION

As biological complexity increases and their genome sizes have increased, organisms have acquired non-coding regions in their genomes (Taft and Mattick, 2003). In the human genome, the ratio of non-coding DNA to total genomic DNA is approximately 98.5%. Recent studies have revealed that transcription is not restricted to protein-coding regions, but occurs throughout the genome (>90%), including non-coding regions. This yields large numbers of non-coding RNAs (ncRNAs; Birney et al., 2007). Increasingly, functional roles for these ncRNAs are being identified. Therefore, the discovery of these abundant ncRNAs should expand our understanding of the mechanisms underlying many biological processes.

Non-coding RNAs are mainly classified as housekeeping or regulatory ncRNAs. Housekeeping ncRNAs include transfer RNAs (tRNAs), ribosomal RNAs (rRNAs), and spliceosomal RNAs. These housekeeping ncRNAs are usually expressed constitutively in cells and are necessary for vital cellular functions. Regulatory ncRNAs are specifically expressed during certain developmental stages, and in certain tissues or diseases. Based on transcript size, regulatory ncRNAs can be further grouped into two subclasses: small ncRNAs (20–200 nt), and long ncRNAs (lncRNAs, >200 nt). MicroRNAs (miRNAs) represent a type of small ncRNA. miRNAs are involved in biological processes such as development, differentiation, and diseases (e.g., cancer) via gene silencing, establishing their biological significance (Mattick and Makunin, 2005).

Recently, functional analyses of lncRNAs have commenced in this emerging field of molecular biology. Although our current understanding of the functional role of lncRNAs is limited, recent reports have revealed characteristics and novel functions of these molecules. Diverse functions of lncRNAs include their involvement in the integrity of the nuclear structure, regulation of gene expression, chromatin remodeling, transcription, and post-transcriptional processing (Mercer et al., 2009; Wilusz et al., 2009). An example of this is the lncRNA NEAT1, which is essential for the organization of paraspeckle structure (Clemson et al., 2009; Sasaki et al., 2009). Xist and Kcnq1ot1 are examples of lncRNAs involved in chromatin remodeling, where they recruit the polycomb complex to the X chromosome or the Kcnq1 domain, respectively. They induce heterochromatin formation and repress gene expression (Silva et al., 2003; Pandey et al., 2008). Through regulation of chromatin remodeling, Xist RNA inactivates the X chromosome and Kcnq1ot1 establishes lineage-specific transcriptional silencing patterns. It is assumed that lncRNAs exert their functions by regulating the localization or activity of proteins through interaction with specific RNA binding proteins. Recently, two independent studies focused on the stability of lncRNAs and revealed the relationships between half-lives of lncRNAs and their functions. Interestingly, the half-lives of lncRNAs vary over a similarly wide range to that of mRNAs. Those lncRNAs with a short half-life include known regulatory ncRNAs, while those with a long half-life are involved with housekeeping functions (Clark et al., 2012; Tani et al., 2012). Considering those characteristics and effects on cellular and molecular mechanisms, we cannot exclude the involvement of lncRNAs from future analyses of biological process and disease pathogenesis.

Recently, several lncRNAs have been identified as being cancer-specific. It is possible these lncRNAs can be harnessed as novel biomarkers or therapeutic targets. In this review, we focus on some well-characterized lncRNAs associated with the progression of cancer, namely metastasis-associated lung adenocarcinoma transcript 1 (MALAT1), HOX antisense intergenic RNA (HOTAIR), and antisense non-coding RNA in the INK4 locus (ANRIL).

## METASTASIS-ASSOCIATED LUNG ADENOCARCINOMA TRANSCRIPT 1 (MALAT1)

MALAT1, also known as NEAT2 (nuclear-enriched abundant transcript 2), was the lncRNA that was originally found to be associated with lung cancer (Ji et al., 2003). MALAT1 is an lncRNA of more than 8000 nt. In the human genome, *MALAT1* is located on chromosome 11q13, a region known to be relevant to tumorigenesis and metastasis. Ji et al. (2003) found that MALAT1 was overexpressed in early-stage metastasizing non-small cell lung cancer (NSCLC). High expression levels of MALAT1 correlated with poor prognosis in NSCLC patients. Therefore, MALAT1 has been proposed as a prognostic marker for metastasis and NSCLC patient survival.

MALAT1 has several unique features. Intracellularly, MALAT1 is stably retained in the nucleus (Miyagawa et al., 2012). We have previously found that the half-life of MALAT1 RNA is around 7.6 h, classifying it as a long-lived non-coding transcript (Tani et al., 2012). The 3′ end of the MALAT1 transcript is cleaved by tRNA processing machinery to yield a tRNA-like small RNA, known as MALAT1-associated small cytoplasmic RNA (mascRNA); however, it localizes to the cytoplasm, has a relatively short half-life, and its function remains unknown (Wilusz et al., 2008). After processing in the nucleus, mascRNA is exported to the cytoplasm, while the abundant and stable MALAT1 long transcript remains in the nucleus.

In the nucleus, MALAT1 specifically localizes to nuclear speckles; subnuclear structures that are enriched for pre-mRNA splicing factors (Hutchinson et al., 2007). Several studies have shown that nuclear speckles function as storage, assembly and modification compartments, supplying splicing factors to active transcription sites (Lamond and Spector, 2003). MALAT1 has been shown to regulate alternative splicing by modulating the distribution and levels of active pre-mRNA splicing factors (SR proteins) (Tripathi et al., 2010). This is the first report showing that MALAT1 directly associates with SR proteins, which are enriched in nuclear speckles, and regulates gene expression post-transcriptionally. Unlike NEAT1 lncRNA, which has been shown to be critical for the integrity of paraspeckles, MALAT1 is not essential for the integrity of nuclear speckles. Depletion of MALAT1 does not affect the localization of nuclear speckle markers (Clemson et al., 2009). MALAT1 appears to be specifically involved in gene regulation by modulating the association of SR proteins with speckles.

**FIGURE 1:**
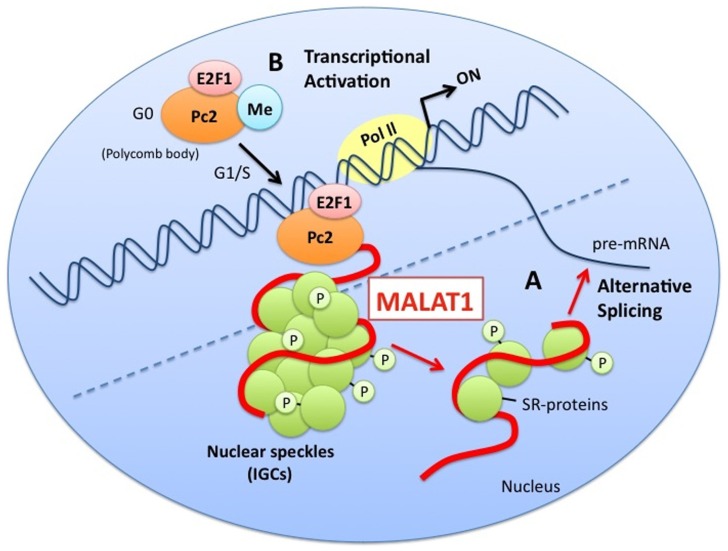
**A model for the functions of MALAT1**. MALAT1 stably localizes to nuclear speckles (interchromatin granule clusters; IGCs). **(A)** MALAT1 regulates alternative splicing by interacting with pre-mRNA splicing factors (SR proteins) and modulating the distribution and levels of active SR proteins. **(B)** MALAT1 regulates relocation of growth-control genes from the repressive environment of polycomb bodies (PcGs) to the gene activation milieu of the interchromatin granules (ICGs), in response to growth signals, by interacting with unmethylated Pc2. This leads to the promotion of E2F1 SUMOylation, and the activation of transcription of genes related to the control of growth.

MALAT1 is also involved in regulated transcriptional programs (Yang et al., 2011a). It has been shown to regulate relocation of growth-control genes from the repressive environment of polycomb bodies (PcGs) to the gene activation milieu of interchromatin granules (ICGs) in response to growth signals by interacting with unmethylated Pc2. This leads to the promotion of E2F1 SUMOylation and activation of transcription for genes associated with growth control. Thus, MALAT1 is thought to have a variety of functions, especially in gene regulation (**Figure 1**).

Although MALAT1 is highly conserved among mammals and is broadly expressed in normal mouse and human tissues (Ji et al., 2003), it is not essential for mouse development (Nakagawa et al., 2012). MALAT1 might play key roles under certain types of external stimuli. MALAT1 is overexpressed not only in lung cancer, but also in breast, pancreas, colon, prostate, and liver cancers (Lin et al., 2007) (**Table 1**), implying key roles in cancer progression. Our group suggested that MALAT1 promotes lung cancer cell motility, which is important for metastasis, through regulation of motility-related genes such as *CTHRC1*, *CCT4*, *HMMR*, and *ROD1*, transcriptionally and/or post-transcriptionally (Tano et al., 2010). Schmidt et al. (2011) showed that decreased expression of MALAT1 in NSCLC xenografts led to impaired tumor formation and growth. These reports suggested that MALAT1 is a potent player in the metastasis process. In other types of cancer cells, such as CaSki cervical cancer cells, MALAT1 is involved not only in cell migration, but also in cell growth and cell cycle progression (Guo et al., 2010). Another report demonstrated that MALAT1 promotes epithelial-to-mesenchymal transition (EMT) by activating the Wnt/β-catenin pathway in bladder cancer cells (Ying et al., 2012). These reports support the hypothesis that MALAT1 is an activator for metastasis and affects metastatic processes. Further investigations are necessary to determine the precise mechanism(s) of metastatic progression effected by MALAT1.

**Table 1:** Cancer-specific long non-coding RNAs (lncRNAs).

lncRNA	Cancer type	Function
MALAT1	Lung cancer (Ji et al., 2003; Schmidt et al., 2011) Breast, pancreas, colon, prostate, liver carcinoma (Lin et al., 2007) Cervical cancer (Guo et al., 2010) Bladder cancer (Ying et al., 2012)	Alternative splicing (Tripathi et al., 2010) Gene relocation (Yang et al., 2011a)
HOTAIR	Breast cancer (Gupta et al., 2010) Colorectal cancer (Kogo et al., 2011) Hepatocellular carcinoma (Yang et al., 2011b) Gastrointestinal stromal tumor (Niinuma et al., 2012) Pancreatic cancer (Kim et al., 2012)	Chromatin remodeling (Gupta et al., 2010; Tsai et al., 2010)
ANRIL	Breast cancer (Turnbull et al., 2010) Nasopharyngeal carcinoma (Bei et al., 2010) Glioma (Shete et al., 2009; Cunnington et al., 2010) Melanoma (Bishop et al., 2009; Cunnington et al., 2010)	Chromatin remodeling (Yap et al., 2010; Kotake et al., 2011)

*Only those cancer-specific lncRNAs that are described in this review are shown.*

## HOX ANTISENSE INTERGENIC RNA (HOTAIR)

HOTAIR is also known as metastasis-associated lncRNA (Gupta et al., 2010). This is a 2.2-kb transcript. Gupta et al. (2010) first revealed that expression of HOTAIR was increased in primary breast tumors and metastases. They showed a positive correlation among high expression levels of HOTAIR, subsequent metastasis and death. Moreover, HOTAIR promotes invasion of breast carcinoma cells and lung metastasis *in vivo*. These findings indicate that HOTAIR is a powerful predictor of metastasis and poor prognosis.

Ultra-high-density *HOX* tiling array analysis revealed that more than 200 ncRNAs were transcribed from the four human *HOX* loci *(HOXA*–*HOXD)* (Rinn et al., 2007). HOTAIR lncRNA is a *HOX* ncRNA transcribed in an antisense manner from the *HOXC* locus. Rinn et al. (2007) also revealed that HOTAIR induces transcriptional silencing of the *HOXD* locus on chromosome 2 by interacting and recruiting the polycomb repressive complex 2 (PRC2) to the *HOXD* locus. PRC2, comprising histone H3 lysine 27 (H3K27) methylase EZH2, SUZ12, and EED, establishes the repressive H3K27me3 chromatin mark and is involved in developmental gene silencing and cancer progression. Gupta et al. (2010) showed that overexpression of HOTAIR in epithelial cancer cells induced genome-wide re-targeting of PRC2. This led to altered H3K27 methylation and gene expression. Genes that were repressed by HOTAIR included tumor suppressor genes, such as cell adhesion molecules of the protocadherin (PCDH) family and JAM2. Repressions of those genes were suppressed by concomitant PRC2 depletion, suggesting PRC2 dependency. This regulation might be involved in the mechanism by which HOTAIR promotes cancer metastasis. Kogo et al. (2011) found that there was a close correlation between HOTAIR expression and PRC2 occupancy in patients with colorectal cancer who have metastases and poor prognosis. Their report also supported the idea that HOTAIR reprograms the chromatin state *via* regulation of PRC2, thereby promoting cancer metastasis.

On the other hand, the *HOXD* region is also bound by CoREST/REST repressor complexes, which contain LSD1, a demethylase that mediates enzymatic demethylation of H3K4me2. Subsequent analysis revealed that HOTAIR also binds to the LSD1/CoREST/REST complex (Tsai et al., 2010). A 5′ domain of HOTAIR binds to PRC2, whereas a 3′ domain of HOTAIR binds to the LSD1 complex. Thus, HOTAIR is a modular bifunctional lncRNA that has distinct binding domains for a histone methylase and a demethylase. It can be assumed that HOTAIR serves as a scaffold for at least two distinct histone modification complexes. The bifunctional role of HOTAIR may be required for coordinating histone modifications in gene silencing to promote metastatic processes.

Numerous studies have suggested that high expression levels of HOTAIR in many types of cancer potentially correlate with metastasis and poor prognosis (Kogo et al., 2011; Yang et al., 2011b; Kim et al., 2012; Niinuma et al., 2012). In a recent study by Kim et al. (2012), it was demonstrated that HOTAIR is not only a prognostic factor, but also a pro-oncogenic factor in pancreatic cancer. Further studies should reveal the functions of HOTAIR during oncogenesis.

## ANTISENSE NON-CODING RNA IN THE INK4 LOCUS (ANRIL)

ANRIL was first identified following genetic analysis of familial melanoma patients with neural system tumors who had a large germline deletion of the entire *INK4B-ARF-INK4A* gene cluster (Pasmant et al., 2007). *INK4B-ARF-INK4A* gene cluster occupies a 42-kb stretch on chromosome 9p21 in humans. This gene locus is homozygously deleted or transcriptionally silenced in a wide range of cancers, with an estimated frequency of approximately 40%, representing one of the most frequently altered genes in human cancer. This locus encodes two cyclin-dependent kinase inhibitors (p15^INK4B^ and p16^INK4A^) and a regulator of the p53 pathway (p14^ARF^). The tumor suppressors p15^INK4B^, p16^INK4A^, and p14^ARF^ are frequently disabled in human cancers, including familial cutaneous malignant melanoma. They inhibit cell cycle progression and influence key physiological processes, such as replicative senescence, apoptosis, and self-renewal of stem cells (Gil and Peters, 2006). The *INK4B-ARF-INK4A* locus is regulated by PRCs and ANRIL is reported to be involved in silencing of this locus.

*ANRIL* spans a region of 126.3 kb, and is transcribed as a 3.8-kb lncRNA in the opposite direction from the *INK4B-ARF-INK4A* gene cluster (Pasmant et al., 2007). *ANRIL* has 19 exons, with two exons of *INK4B* overlapping. The first exon of *ANRIL* is located -300 bp upstream of the transcription start site of *ARF*. A stronger positive correlation exists between the expression of *ANRIL* and *ARF* in normal human tissue and tumors. These results suggest that these two genes share a bidirectional promoter under physiological and pathological conditions. Several reports have demonstrated that the ANRIL transcript is processed as alternative splice variants, including a circular form of ANRIL RNA (Folkersen et al., 2009; Burd et al., 2010).

Recent genome-wide association studies have identified *ANRIL* as a risk locus for several cancers, including breast cancer, nasopharyngeal carcinoma, basal cell carcinoma, and gliomas (Shete et al., 2009; Stacey et al., 2009; Bei et al., 2010; Turnbull et al., 2010; Pasmant et al., 2011). Many single-nucleotide polymorphisms (SNPs) have been identified in the *ANRIL* locus. These are highly associated with the expression of ANRIL. Cunnington et al. (2010) revealed that the glioma risk allele SNP rs1063192-C is highly correlated with increased ANRIL expression, while the melanoma risk variant SNP rs1011970-T correlated with decreased expression of ANRIL. Chromosome 9p21 SNPs are known to correlate with susceptibility to glioma and malignant melanomas (Bishop et al., 2009; Shete et al., 2009; Wrensch et al., 2009). Other SNPs in the *ANRIL* locus have also been reported to be associated with its expression and involvement in susceptibility to coronary artery disease (Liu et al., 2009). Although the precise molecular mechanism(s) by which ANRIL influences cancer progression is largely unknown, it is assumed that SNPs modulate its expression and that this may lead to susceptibility to cancers.

Recently, two reports have provided mechanistic insights into the molecular functions of ANRIL during epigenetic transcriptional repression. Kotake et al. (2011) reported that ANRIL binds to SUZ12 and recruits PRC2 to repress expression of the *p15*^*INK*4*B*^ locus. Depletion of ANRIL caused defects in SUZ12 binding to the *p15*^*INK*4*B*^ locus, increased the expression of *p15*^*INK*4*B*^, but not *p16*^*INK*4*A*^ or *p14*^*ARF*^, and inhibited cellular proliferation. Yap et al. (2010) showed that ANRIL binds to chromobox 7 (CBX7) within PRC1 and recruits it to the *INK4B-ARF-INK4A* gene locus to control the expression levels of the genes located at this locus, leading to regulation of cellular senescence. These studies on ANRIL suggest that it has bifunctional roles, serving as a scaffold for both PRC1 and PRC2, as shown for HOTAIR. Further studies are necessary to elucidate the detailed mechanism(s) regarding epigenetic transcriptional regulation by ANRIL and to reveal its role in cancer progression.

## CONCLUSIONS AND PERSPECTIVES

Cancer is a complex disease, involving various changes in gene expression. These gene expression changes cause cancer development, including metastasis, by affecting cell proliferation, invasion, and angiogenesis. Although large numbers of protein-coding genes have been shown to affect cancer progression and several mechanisms that alter the ability to promote cancer progression have been identified, the molecular determinants of cancer progression remain unknown. ncRNAs have provided a new insight into cancer biology and provided several lines of evidence to suggest there is a correlation between their expression and promotion of cancers. In addition to the lncRNAs described above, increasing numbers of lncRNAs have been identified as being associated with cancer, such as αHIF (antisense hypoxia-inducible factor-1α; Span et al., 2011), HULC (highly upregulated in liver cancer; Wang et al., 2010), and PCGEM1 (prostate specific gene 1; Petrovics et al., 2004). Most of these lncRNAs are specifically expressed in certain tumors, at different stages of tumor progression, or in various tissues. These characteristics would suggest that lncRNAs could represent biomarkers for diagnosis and prognosis, in accordance with distinct cancer types. In addition, lncRNAs whose expression is associated with poor prognosis might be potential therapeutic targets for gene therapy, such as by siRNAs. For example, the inhibition of MALAT1, which is associated with metastasis and poor prognosis, might be an effective therapeutic strategy for metastatic cancer, without affecting normal cells because it is not necessary for the viability of normal cells. Recently, Wheeler et al. (2012) reported a novel strategy to eliminate MALAT1 effectively *in vivo* using antisense oligonucleotides (ASOs). This result strongly supports the high potential of curing metastatic cancers by the elimination of MALAT1 using gene therapy.

Several reports have suggested that lncRNAs can act as key factors in the regulation of gene expression; therefore, lncRNAs can potentially uncover the molecular mechanisms underlying the development of cancer, thereby leading to new strategies for cancer therapy. For example, determination of both binding proteins associated with each lncRNA and the genes regulated by lncRNAs would reveal the molecular mechanisms underlying cancer development by lncRNAs. Moreover, it is possible that not only lncRNAs, but also both binding proteins and the target genes regulated by lncRNAs, could be novel therapeutic targets, which may lead to the development of novel cancer treatments. The functional analysis of lncRNAs related to cancer represents a major task that should be carried out in the near future.

## Conflict of Interest Statement

The authors declare that the research was conducted in the absence of any commercial or financial relationships that could be construed as a potential conflict of interest.
